# The association between asymptomatic and mild neurocognitive impairment and adherence to antiretroviral therapy among people living with human immunodeficiency virus

**DOI:** 10.4102/sajhivmed.v19i1.674

**Published:** 2018-04-12

**Authors:** Violet Awori, Peter Mativo, Gerald Yonga, Reena Shah

**Affiliations:** 1 Department of Medicine, Aga Khan University Hospital, Kenya

## Abstract

**Background:**

Asymptomatic cognitive impairment in human immunodeficiency virus (HIV)-infected patients has recently been recognised as part of HIV-associated neurocognitive disorders. This has been implicated as one of the causes of poor adherence to antiretroviral therapy (ART).

**Objective:**

To assess the association between neurocognitive impairment (asymptomatic and mild forms) and adherence to ART.

**Methods:**

This was a cross-sectional survey involving 218 participants consecutively sampled from those attending the HIV treatment clinic at Aga Khan University Hospital in Nairobi. Data collected included quantitative primary data on pre-defined baseline characteristics, neurocognitive assessment by Montreal Cognitive Assessment (MoCA) tool (Appendix 1), instrumental activities of daily living by Lawton score and objective and subjective adherence measures by medication possession ratio (MPR) and simplified medication adherence questionnaire (SMAQ) (see Appendix 2). Univariate and bivariate analyses were conducted to determine the strengths of association between predictor and the outcome variables.

**Results:**

Among the 218 participants in the study, a total of 69% had asymptomatic to mild neurocognitive impairment as assessed by the MoCA tool, while a total of 66% were determined as being adherent to ART by objective measures (by MPR) compared to subjective rates of 77% as assessed by SMAQ. However, no statistically significant association was observed between the presence of asymptomatic or mild neurocognitive impairment and likelihood of adherence to ART (p > 0.05).

**Conclusion:**

Even though asymptomatic and mild forms of cognitive impairment are prevalent in the population studied, there was no significant association between cognitive impairment and adherence to treatment.

## Introduction

Human immunodeficiency virus (HIV) is associated with neurological complications, including neurocognitive impairment. The early screening neuropsychological tests were designed to assess cortical functions.^1,2^

The widely used screening tests, such as the International HIV Dementia Scale (IHDS), mainly assess cortical function and are not sensitive to the milder forms of neurocognitive impairment. HIV-associated neurocognitive disorder (HAND) has, however, been shown to mainly affect subcortical functions.^3^

Adherence is one of the major determinants of successful viral suppression among people living with HIV (PLWHIV) on antiretroviral therapy (ART).^4^ Mbugua found out that in the HIV-positive patients attending the HIV clinic, 52% of the patients had an adherence rate of 95% or more, while 48% had suboptimal adherence, which was defined as an adherence less than 95%.^4^ Advisory documents indicate that an adherence rate of 95% is necessary for successful viral suppression.^5,6^ There are many causes of poor adherence, some of which are attributed to neurocognitive impairment that may be asymptomatic or mild, and thus go clinically unrecognised if not assessed.^3^

The American Academy of Neurology (AAN) has identified two forms of HAND since 1991, namely minor cognitive motor disorder (MCMD) and HIV-associated dementia (HAD).

The HIV Neurobehavioral Research Centre (HNRC) modified the criteria, to include an additional group, and these criteria were adopted by the AAN in 2007.^7^ The modified criteria, also known as the Frascati criteria, recognised the following three conditions: asymptomatic neurocognitive impairment (ANI), HIV-associated mild neurocognitive disorder (MND) and HAD. The notable addition was ANI, as research has shown a considerable number of HIV patients with clinically demonstrable cognitive abnormality but no impairment of function.^7^ Asymptomatic neurocognitive impairment refers to the patients with acquired cognitive impairment in at least two ability domains, but the impairment does not interfere with everyday functioning. MND refers to acquired cognitive impairment involving at least two ability domains, with mild interference in daily functioning either by self-report or observation by others. HIV-associated dementia refers to marked acquired cognitive impairment with marked interference in day-to-day functioning.^7^ ANI and MND are the mild forms of HAND.

## Literature review

Human immunodeficiency virus is a neurotropic infection and invades the central nervous system from the time of primary infection.^8^

The introduction of ART heralded a significant change in the spectrum of HAND, with prevalence of HAD decreasing and the prevalence of the milder forms of HAND increasing, predominantly ANI. Therefore, relying on self-report, as was the case previously, missed out this population.^7^

In a study carried out in Botswana among HIV-positive patients attending HIV clinic to assess the neurocognitive impairment, it was found that despite 97.5% of the patients being on ART, 38% of the population had neurocognitive impairment as assessed by the IHDS. According to this study, more than one-third of the population studied had neurocognitive impairment despite being on ART for more than two years, and one of the reasons for this finding was attributed to the likely poor adherence to ART.^9^

Another study carried out at Kenyatta National Hospital (KNH) in 2011 utilised IHDS to assess cognitive function and determined the prevalence of neurocognitive impairment in HIV-positive patients to be 26%, of which 23% were not on ART.^10^

A meta-analysis of studies on prevalence of HAND in HIV-positive patients on ART for more than six months (data collected up to June 2012, and using the IHDS) in seven countries in sub-Saharan Africa (Uganda, Zambia, Malawi, Botswana, Nigeria, Cameroon and South Africa) concluded that the prevalence of neurocognitive impairment was more than 30%.^11^

The CNS HIV Antiretroviral Therapy Effects Research (CHARTER) Study is a landmark longitudinal prospective study that was carried out in the ART era and as illustrated below, the most prevalent form of HAND was found to be the ANI followed by MND.^12^

The IHDS has been shown to be effective in screening for HAD; however, it is not accurate for screening for mild cognitive impairment as it either over-reports or under-reports impairment. The IHDS assesses only cortical function and thus can miss out patients with subcortical dysfunction, as in HAND. Over-reporting can be caused by human error among people administering the IHDS.^13,14,15,16^

The Montreal Cognitive Assessment (MoCA) tool used for assessing subcortical function has shown promise in HIV mild cognitive impairment.^15,17^ It is a 10-min test that is ideal as a screening test.

The MoCA has been validated and widely used in many countries, in non-HIV populations, to screen for milder forms of cognitive impairment. With a cut-off less than 26 for mild neurocognitive impairment, the sensitivity was 83% and the specificity was 81%.^18^ However, in PLWHIV, different authors used scores of either 26 or 27, based on their local validation studies. Chan et al. used MoCA in a study where a diagnosis of HAND was made in 22.7% of 132 HIV-positive patients in Singapore. In the study, Chan stated that local validation studies had a cut-off score of 26 or 27 out of 30, even though the studies were still under peer review.^17^ In a study carried out on 100 HIV-positive patients to screen for neurocognitive impairment with the use of MoCA as a screening tool, it was found to have a sensitivity of 85%, specificity of 40%, negative predictive value of 48% and a positive predictive value of 81%, with a cut-off score of < 26.^19^ Raising the cut-off point of the MoCA was shown to increase sensitivity but reduce specificity.^3,20^ Making a diagnosis of HAND according to Frascati criteria,^7^ which was adopted by the AAN in 2007, requires the use of a neuropsychological battery of tests, as well as comparison with normative data from the population. The MoCA tool attempts to assess the eight domains of cognition to help in screening of patients who would then be referred to neuropsychologists for definitive diagnosis. These domains include visuospatial or executive functioning, naming, attention, concentration and calculation, language, abstraction, delayed recall and orientation. In this study, the MoCA was utilised for screening for cognitive impairment, with a cut-off score less than 26 defined as cognitive impairment.

The Lawton Instrumental Activities of Daily Living (IADL) assesses the eight domains of functions and is most useful for identifying how a person is functioning at the present time (Appendix 3). These domains include the ability to use a telephone, shop, do housekeeping, complete laundry work, transport, and the responsibility for taking medication and to handle finances. The validity was tested by determining its correlations with four scales that measured assessment of functional status and all correlations were found to be significant at 0.01 or 0.05 level.^21^ The tool was also used by Chan^17^ in evaluation of HAND in the population of 132 HIV-positive patients. High scores depict independence, while low scores depict high levels of dependence.

The World Health Organization (WHO) recommends ART adherence of > 95% to achieve the best treatment outcomes.^5,6^

### Justification

The HIV prevalence in Kenya is 5.6% according to the Kenya AIDS Indicator Survey of 2012 (KAIS 2012) report released in 2014.^22^ About 84.5% of HIV-positive patients who are eligible for ART were on treatment according to the 2012 survey. The adherence rates are still suboptimal as shown by a study carried out at Aga Khan University Hospital in 2011, which showed that only 52% of the population had adherence rates above 95% despite 82% of the patients self-reporting an adherence rate of more than 95%.^4^

Although there has been a decline in the incidence of HAD in the ART era, the prevalence rate of HAND remains elevated.^23^ Cognitive assessment is not routinely assessed in our HIV clinics and is also not recommended in current Kenyan treatment guidelines.

Poor judgement, memory impairment and poor decision-making as components of cognition may lead to poor adherence rates in HIV-positive patients who are on ART.^24,25^ One primary cause of treatment failure has been linked to HIV resistance, related to suboptimal adherence rates to ART.^2^ Cognitive impairment may lead to failure to maintain a stable income-generating activity as well as poor decision-making in lifestyle choices, such as taking medication.^3^

Patients, who are HIV-positive with other comorbid conditions, will be required to take several medications, and compliance to these medications requires good cognition.

In patients with drug resistance requiring complex regimens or those taking concurrent treatment for tuberculosis or *Pneumocystis jirovecii* pneumonia, poor cognition may lead to further treatment failure.

Early recognition of neurocognitive impairment will also guide clinicians towards appropriate referrals and further tests for patients as well as strengthening of adherence counselling.

Knowledge of how neurocognitive impairment affects ART adherence will therefore significantly contribute to guidelines in dealing with this potential factor in improving outcomes for PLWHIV.

### Primary objective

To determine the association between the milder forms of cognitive impairment (ANI and MND) and adherence to ART in PLWHIV attending clinic at Aga Khan University Hospital, Nairobi.

## Methodology

### Study design

The study is an analytical cross-sectional survey assessing the association between mild cognitive impairment (asymptomatic and MND) and adherence to treatment with ART in HIV-positive patients.

### Study setting and period

The study was carried out in the HIV clinic at Aga Khan University Hospital (AKUHN), situated in Nairobi, Kenya.

About 635 HIV-positive patients are on follow-up at the AKUHN’s infectious diseases clinics, out of which an estimated 95% (603) are on treatment with ART (about 500 [83%] of them for at least six months, hence eligible for this study). The study was conducted between October 2015 and May 2016, a period during which all those eligible for the study visited the clinic at least once and had a chance to be selected to participate.

### Sample size calculation

The sample size for this study was derived using a sampling formula for a finite population, with an error margin no larger than 0.05.^26^ This is as shown below:

$$n=\frac{m}{1+\frac{m-1}{N}}$$ [Eqn 1]

where

$$m=\frac{Z_{\alpha /2}^{2}p(1-p)}{d^{2}}$$ [Eqn 2]

So that:

- Z_α__/2_ = standard normal variate (1.96) for level of significance (0.05);
- *N* = total number of population, which is 500;
- *n* = sample size;
- *m* = sample size necessary for estimating the prevalence of the total population, *N*;
- *d* = error, 0.05;
- *p* = prevalence of non-adherence in HIV-positive patients attending clinic, estimated at 48% from a previous study conducted in the same population^4^;
- $m=\frac{1.96^{2}*0.48(1-0.48)}{0.05^{2}}(\text{m}=383.54)$;
- Therefore, $n=\frac{383.54}{1+\frac{383.54-1}{500}}(\text{n}=218\text{ patients})$.

### Inclusion criteria

- ≥18 years of age and ≤65 years;
- HIV-positive on ART for over six months;
- Ability to read and write in English.

### Exclusion criteria

- Patients with an opportunistic infection affecting the CNS in the last 12 months or non-CNS infection in the last three months, or known to have a mental illness such as depression or schizophrenia or having epilepsy.
- History of current substance abuse.
- History of head injury with loss of consciousness exceeding 30 min.

### Sampling technique and data collection

Over the study period, a total of 635 patients attended the clinics, of whom, 500 were eligible for the study.

Consecutive sampling was utilised, recruiting every eligible clinic patient into the study as they visited the clinic during this period, until the sample size was met.

The following is the recruitment procedure:

- HIV-positive patients were recruited from the HIV clinic at AKUHN by the principal investigator during opening hours (Monday to Saturday).
- Informed consent was obtained from the patients who had met the eligibility criteria. The principal investigator discussed the consent process with the patients in detail and they were given a chance to decline participation.
- The patients were provided with a questionnaire to obtain biographical data.
- The MoCA questionnaire was administered to patients to assess for cognitive function, with a cut-off less than 26.
- The patient’s functional status at work and at home was assessed using the Lawton’s IADL tool, by self-reporting.
- Self-reported adherence to ART using the simplified medication adherence questionnaire (SMAQ) tool was also established.
- Medication possession ratio (MPR) was then calculated based on the pharmacy dispensing records for about three – eight months to assess their possession rate as an objective measure of adherence using the following formula:
  $$\frac{\text{Pills dispensed}/\text{pills prescribed per day}}{\text{Number of days between refills}}\times 100\%.$$ [Eqn 3]

The MPR is a validated, pharmacy-based method for assessment of adherence in areas where pill count is not normally done as is the case at the Aga Khan University Hospital, Nairobi.^27^

For a diagnosis of HAND to be made, the Frascati criteria, adopted by AAN, need to be met; however, for screening purposes, normal cognition is any score of 26 and above. The MoCA cannot distinguish between HAD and minor forms of neurocognitive impairment. However, as per the Frascati criteria, low cognitive scores, together with increased dependence on others for IADL, would give a diagnosis of HAD.^7^

Optimal adherence was defined as 95% and above, as per the WHO’s recommendations, with suboptimal adherence being any value less than 95%.^5,6^ The MPR was calculated for each patient based on the pharmacy dispensing records, for at least three months.

The SMAQ was utilised for assessment of self-reported adherence for comparison with the MPR, and it has been validated for use in the HIV-positive population, with a sensitivity of 72% and a specificity of 91%.^28,29^

### Data analysis

This study utilised a quantitative design involving collection and analysis of quantitative primary data about pre-defined variables. Additional data were also collected on other patient-specific attributes that are likely to affect adherence patterns.

Descriptive statistics were used to summarise the data including frequency tables for demographic characteristics of the participants. Other baseline characteristics related to the participants’ HIV treatment were also analysed.

The patients were then grouped based on their neurocognitive status (with and without impairment).

Other baseline factors with potential to affect adherence in the two patient groups were also compared. Thereafter, bivariate analysis using chi-square test was used to test the relationship between individual predictor and outcome variables. The choice of chi-square test in this regard is especially suited to conducting hypothesis tests for categorical data such as that used for this study.

Data collected, collated and cleaned in Excel worksheets were eventually analysed using Statistical Package for Social Sciences (SPSS) version 21.

## Ethical consideration

The study was carried out with approval from the Department of Internal Medicine, AKUHN and the Ethical and Scientific Review Committee. Informed written consent was obtained from all participants.

## Results

A total of 500 patients were found to be eligible for the study. A final sample size of 218 patients was recruited to participate in the study. For those not eligible, the main reasons for ineligibility were duration on ART of less than six months, presence of space occupying lesions and other central nervous system abnormalities, and extremes of age above 65 years. No patients were found to have HAD, 69% had minor neurocognitive dysfunction and 31% had no neurocognitive dysfunction. Those selected underwent adherence assessment. The overall optimal adherence rate for this population was 66% by objective assessment (MPR) and 77% by subjective assessment (SMAQ).

### Summary of baseline characteristics

Of the 218 participants selected for the study, a total of 215 completed the MoCA neurocognitive assessment. The study population varied according to different baseline demographic and HIV treatment-related characteristics (Tables 1 and 2).

**TABLE 1:** Baseline demographic characteristics.

Baseline characteristic	Variable	MoCA neurocognitive assessment score			
		Normal	%	Impaired	%
Overall outcome	-	67	31	147	69
Age	Mean	42.3 years	-	45.5 years	-
Gender	Male	34	51	67	46
	Female	33	49	80	54
Education level	Primary	1	2	11	8
	Secondary	9	13	29	20
	Tertiary	57	85	106	73
Marital status	Married	37	55	101	69
	Widowed	11	16	14	10
	Divorced or separated	4	6	10	7
	Single	15	22	21	14
Living with family	Yes	57	85	127	86
	No	10	15	20	14
Employment status	Employed	48	72	96	66
	Self-employed	15	22	40	27
	Unemployed	4	6	10	7
Level of income (US dollars per month)	0–100	5	8	22	15
	110–500	12	19	46	32
	510–1000	20	32	31	22
	> 1000	26	41	45	32

MoCA, Montreal Cognitive Assessment.

**TABLE 2:** Human immunodeficiency virus treatment-related characteristics.

Characteristic	Variable	MoCA score			
		Normal	Normal (%)	Impaired	Impaired (%)
Duration since HIV diagnosis (years)	Mean	7.6 years	-	8.0 years	-
Duration on ART (years)	Mean	5.47 years	-	6.69 years	-
Source of ART medicines	AKUHN	57	85	128	88
	Other	10	15	18	12
Payment for medication	Cash	18	27	41	28
	Insurance	43	64	95	65
	Insurance/cash	1	1	5	3
	Free	5	7	5	3
ART regimen	EFV-based	48	72	98	70
	PI-based	11	16	32	23
	Other	8	12	10	7
First viral load	Suppressed	51	88	103	82
	Not suppressed	7	12	22	18
Latest viral load	Suppressed	41	68	84	67
	Not suppressed	19	32	42	33

AKUHN, Aga Khan University Hospital, Nairobi; ART, antiretroviral therapy; HIV, human immunodeficiency virus; MoCA, Montreal Cognitive Assessment; EFV, Efavirenz; PI, Protease Inhibitor.

Those with normal neurocognitive function had a mean age of 42.3 years, while the mean age for those with impaired cognitive function was 45.5 years.

The source of a patient’s medicine was thought to be important, as it determined the availability of the medicine and therefore adherence to treatment plans. This factor also determined how many patients could be assessed objectively by MPR owing to availability of pharmacy records. Similarly, income and therefore ability to afford medication was important, as it related to adherence to treatment and outcomes thereafter.

#### Bivariate analysis: Cognitive impairment and adherence to antiretroviral therapy (as measured by medication possession ratio)

A bivariate analysis was conducted to determine the existence of any significant association between the presence of mild cognitive impairment as measured by the MoCA score and the ability to adhere to prescribed treatment with ART as measured objectively through calculation of the MPR. The results showed a lack of significant association (*p* = 0.490) between the two variables (Table 3).

**TABLE 3:** Bivariate analysis of Montreal Cognitive Assessment scores and Medication Possession Ratio.

Neurocognitive assessment	Variable	Adherence by MPR		*p*
		Optimal (number of participants)	Suboptimal (number of participants)	
MoCA neurocognitive assessment score	Normal	34	15	0.490
	Impaired	74	42	

MPR, medication possession ratio; MoCA, Montreal Cognitive Assessment.

#### Bivariate analysis: Cognitive impairment and adherence to antiretroviral therapy (as measured by simplified medical adherence questionnaire)

A bivariate analysis was conducted to determine the existence of any significant association between the presence of mild cognitive impairment as measured by the MoCA score and the ability to adhere to prescribed treatment with ART as measured subjectively through the SMAQ questionnaire. The results showed a lack of significant association (*p* = 0.658) between the two variables (Table 4).

**TABLE 4:** Bivariate analysis of Montreal Cognitive Assessment and simplified medication adherence questionnaire.

Neurocognitive assessment	Variable	Adherence by SMAQ		*p*
		Adherent (no. of participants)	Non-adherent (no. of participants)	
MoCA neurocognitive assessment score	Normal	50	17	0.658
	Impaired	113	33	

MoCA, Montreal Cognitive Assessment; SMAQ, simplified medication adherence questionnaire.

### Secondary analysis

Secondary analysis was conducted for any association between baseline patient characteristics and the observed adherence patterns; as such, characteristics could serve as confounders to the outcome variable. Table 5 shows the outcomes of the bivariate analysis between selected baseline characteristics of participants and the adherence patterns as assessed objectively by MPR and subjectively by SMAQ.

**TABLE 5:** Bivariate analysis for baseline characteristics and adherence.

Variable	Category	Adherence by MPR (no. of participants)			Adherence by SMAQ (no. of participants)		
		Optimal	Suboptimal	*p*	Adherent	Non-adherent	*p*
Sex	Male	55	28	0.870	78	22	0.633
	Female	54	29		85	28	
Education level	Primary	8	2	0.443	9	3	0.918
	Secondary	24	10		30	8	
	Tertiary	77	45		123	39	
Marital status	Married	72	39	0.857	112	26	0.077
	Widowed	10	7		19	6	
	Divorced or separated	8	3		8	6	
	Single	18	8		23	12	
Living with family	Yes	91	51	0.298	143	41	0.301
	No	18	6		20	9	
Employment status	Employed	75	33	0.348	115	28	0.079
	Self-Employed	27	20		39	16	
	Unemployed	7	4		8	6	
Payment mode	Cash	30	19	0.195	46	13	0.879
	Insurance	75	35		106	31	
	Free	2	0		7	3	
	Insurance or cash	1	3		4	2	
Source of medicines	AKUHN	104	55	0.949	141	43	0.850
	Other	4	2		21	7	

MPR, medication possession ratio; SMAQ, simplified medication adherence questionnaire.

The numbers do not consistently add up owing to some missing data. An example is MPR calculation owing to patients collecting their medication from outside the hospital.

As shown in Table 5, there was no statistically significant association between any baseline patient characteristics and adherence by either MPR or SMAQ (all *p-*values were > 0.05).

A sub-analysis was conducted to determine the association between the measures of cognitive impairment using MoCA and the latest viral load as a surrogate measure of adherence. With a cut-off of 1000 copies per millilitre (mL), patients with a viral load greater than 1000 copies/mL were considered not suppressed and therefore non-adherent to ART, while patients with a viral load less than 1000 copies/mL were considered to have a suppressed viral load and therefore adherent to ART.^30^ There was no significant association between the presence of cognitive impairment and the treatment outcomes as measured by viral load (*p =* 0.238) (Table 6).

**TABLE 6:** Bivariate analysis with use of the latest viral load as a measure of adherence.

MoCA score assessment	Latest viral load		*p*
	Viral load suppressed (< 1000 copies/mL)	Viral load not suppressed (> 1000 copies/mL)	
Normal	57	3	0.238
Impaired	115	13	
Unknown	30	-	

MoCA, Montreal Cognitive Assessment.

## Discussion of findings

The study was carried out among patients attending the clinic who are on follow-up, and as far as activities of daily living assessment were concerned, they were independent. The population studied had a mean age of 46.5 years in males and 42.7 years in females. This is an older age group as compared with the national statistics according to KAIS, 2012, which shows that HIV prevalence is the highest among women aged 30–39 years.^22^ However, among the males, it is in line with the national statistics which show the highest prevalence between 45 and 49 years of age.

The rate of cognitive impairment in this group is high, which corresponds to some of the studies carried out, such as the CHARTER study, which showed a 52% prevalence of HANDs, with majority being asymptomatic and mild cognitive disorders, and only 2% of this population having HAD. On exclusion of patients with severe confounding comorbidities, 46.9% of the remaining patients received a diagnosis of HAND, and 70% of this population had ANI.^12^ In the current study, there was no patient presumably identified to have HAD, as assessed by both MoCA and Lawton score. One patient reported that she was independent in IADL, even though she had a low MoCA score of 9/30.

The high prevalence of cognitive impairment in this group could be because of several factors such as the MoCA, though validated in other countries, being not wholly culturally appropriate in our circumstances. Most participants had difficulty recalling some unfamiliar items such as daisy, velvet and the word couch, as part of the statement in the language section, which were words that needed to be assessed as part of cognitive assessment. This was also seen in a study by Robbins et al., which was carried out in South Africa among Xhosa-speaking people. They had to translate the MoCA to the Xhosa language as well as modify it for the tool to be culturally acceptable.^3^

The MoCA screening tool was initially designed to screen for mild cognitive impairment by Nasreddine et al. and the cut-off score was set at less than 26.^18^ However, several validation studies in the HIV setting have used different cut-off scores. This could account for the classification of patients as having normal versus impaired cognition, based on the cut-off score set. In settings where a complete neurophysiological battery of tests cannot be done readily, MoCA is a good screening tool with high enough sensitivity in milder forms of cognitive impairment.

The respondents were recruited during clinic hours in the morning. Most of the patients were employed and as they were coming in the routine clinic time, they were either coming from work – night duty, or on their way to work. The sense of spending more time than their usual clinic hours seemed to be a factor in some of their performances, as some patients reported that they were going to be late, were tired and could therefore not concentrate well or were not in the best state of mind to respond to the questions. It is postulated that this could be one of the factors contributing to the high prevalence rate of cognitive impairment. Cognitive assessment is ideally a continuous process. The MoCA tool is designed for screening for cognitive impairment, but definitive diagnosis is done by a neurophysiologic battery of tests, such as the HNRC battery of tests. It would be necessary to carry out the tests several times and get an average score; however, as this was a cross-sectional survey, we only relied on one meeting with the patient to make our assessment. The study design also limited the extent to which causality could be established. The study was also limited by lack of data for patients who obtained their medication or did their laboratory tests outside the hospital.

According to this study, the adherence rate, calculated by MPR, was 66%, compared with 77% as per subjective testing. These results are comparable to a study conducted in AKUHN, Kenya.^4^

Our study had several strengths. This is the first study, to our knowledge, that has been carried out in Kenya, using MoCA as a screening tool for cognitive impairment and associating it with adherence to ART. It therefore gives a prevalence of asymptomatic and mild cognitive impairment in this population, whereas previous studies assessed prevalence of HAND in general, and did not focus on the mild forms of cognitive impairment. The earlier studies also used IHDS and MMSE, which have been found to be less sensitive for subcortical dysfunction that is more dominant in the mild forms of HAND.

The sample size of 218, for a finite population of 500 eligible patients, was adequate for assessment of the association between milder forms of cognitive impairment and adherence to ART; hence, the results may be generalisable in similar settings but should be extrapolated to other populations with caution because of the specific characteristics of the population studied.

## Figures and Tables

**FIGURE 1-A1:**
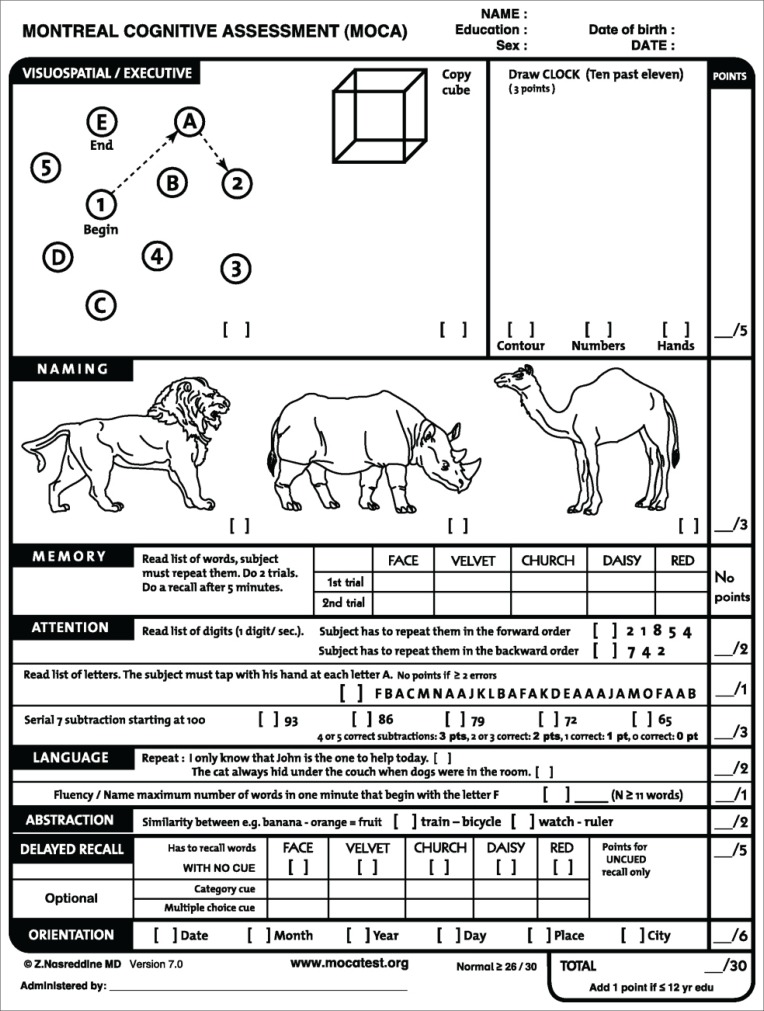
Montreal Cognitive Assessment (MoCA).

**FIGURE 1-A2:**
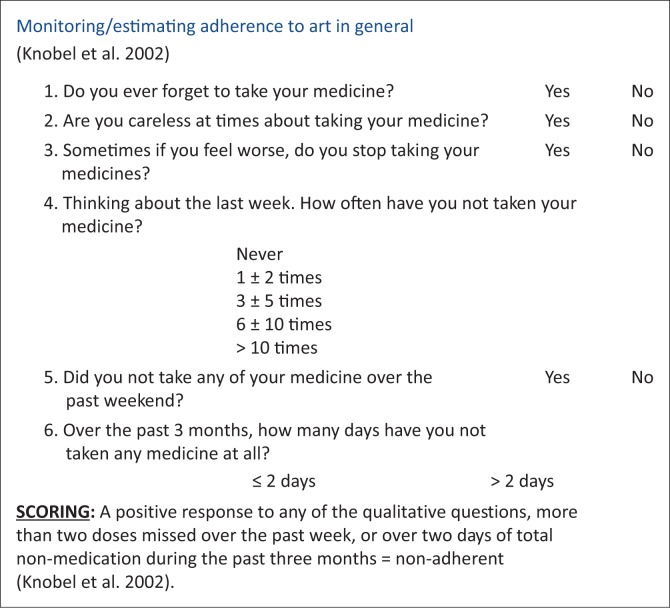
Simplified Medication Adherence Questionnaire (SMAQ)

## Appendix 3

### Lawton’s instrumental activities of daily living

**Table:**

**Date:**	**Name**

Please check the box that most applies for each activity:

**Table:**

Activity	Need no help (2 pts. each)	Need some help (1 pt. each)	Unable to do at all (0 pts. each)
1. Using the telephone	___	___	___
2. Getting to places beyond walking distance	___	___	___
3. Grocery shopping	___	___	___
4. Preparing meals	___	___	___
5. Doing housework or handyman work	___	___	___
6. Doing laundry	___	___	___
7. Taking medications	___	___	___
8. Managing money	___	___	___

**Total Score: ___ =**	**(___ x 2 =) ___ +**	**(___ x 1=) ___ +**	**0**

*Source*: Lawton and Brody.^21^ Copyright (c) by The Gerontological Society of America. Used by permission of the Publisher.
